# Announcements

**Published:** 2015-04-03

## National Youth HIV and AIDS Awareness Day — April 10, 2015

National Youth HIV and AIDS Awareness Day on April 10 is the first awareness day to recognize the specific impact of HIV/AIDS epidemic on young persons. A disproportionate number of new HIV infections occurs among youths (*1*). In the United States, young persons aged 13–24 years accounted for an estimated 26% of all new HIV infections in 2010 (*1*). Nearly 60% of new infections in youths occur in blacks/African Americans, approximately 20% in Hispanics/Latinos, and approximately 20% in whites (*1*). However, the percentage of youths tested for HIV is low compared with other age groups (*1*). Among the estimated 34% of U.S. high school students who are sexually experienced, only 22% have ever been tested for HIV (*2*). The Community Preventive Services Task Force recommends risk reduction interventions in school and community settings to prevent HIV among adolescents (*3*). Individual-level and group-level HIV prevention interventions provide knowledge, skill building, and increased motivation to adopt behaviors that protect against HIV infection, specifically for youths at high risk for HIV.

CDC has a multifaceted approach to meet the goals of the National HIV/AIDS Strategy (*4*), with special emphasis on reducing HIV infection by educating young persons about HIV before they begin engaging in behaviors that place them at risk for infection. CDC biennially collects and reports data on health risk behaviors with the national, state, territorial, tribal government, and local school-based surveys of representative samples of students in grades 9–12.* Through its Act Against AIDS campaign,† CDC provides clear messages about HIV prevention and reducing its stigma, especially for high-risk groups, including young persons. Additionally, CDC funds public health departments, education agencies, and community-based organizations to expand HIV prevention education, behavioral interventions, and health services for young persons.

National Youth HIV and AIDS Awareness Day is a component of CDC’s efforts to 1) prevent HIV, other STDs, and teen pregnancy and promote lifelong health among young persons, and 2) acknowledge and address the needs of young persons related to HIV/AIDS prevention. Additional information regarding youth and HIV/AIDS prevention is available at http://www.cdc.gov/hiv/ and http://www.cdc.gov/healthyyouth/.

## Footnotes

* Available at http://www.cdc.gov/yrbs.

† Available at http://www.cdc.gov/actagainstaids.
